# Accelerated habit formation following amphetamine exposure is reversed by D_1_, but enhanced by D_2_, receptor antagonists

**DOI:** 10.3389/fnins.2013.00076

**Published:** 2013-05-15

**Authors:** Andrew J. D. Nelson, Simon Killcross

**Affiliations:** ^1^School of Psychology, Cardiff UniversityCardiff, UK; ^2^School of Psychology, University of New South WalesSydney, NSW, Australia

**Keywords:** amphetamine, sensitization, D_1_ and D_2_ receptor subtypes, habits, goal-directed

## Abstract

Repeated exposure to the psychostimulant amphetamine has been shown to disrupt goal-directed instrumental actions and promote the early and abnormal development of goal-insensitive habitual responding (Nelson and Killcross, 2006). To investigate the neuropharmacological specificity of this effect as well as restore goal-directed responding in animals with pre-training amphetamine exposure, animals were treated with the non-selective dopamine antagonist α-flupenthixol, the selective D_1_ antagonist SCH 23390 or the selective D_2_ antagonist eticlopride, prior to instrumental training (three sessions). Subsequently, the reinforcer was paired with LiCL-induced gastric-malaise and animals were given a test of goal-sensitivity both in extinction and reacquisition. The effect of these dopaminergic antagonists on the sensitivity of lever press performance to outcome devaluation was assessed in animals with pre-training exposure to amphetamine (Experiments 1A–C) or in non-sensitized animals (Experiment 2). Both α-flupenthixol and SCH23390 reversed accelerated habit formation following amphetamine sensitization. However, eticlopride appeared to enhance this effect and render instrumental performance compulsive as these animals were unable to inhibit responding both in extinction and reacquisition, even though a consumption test confirmed they had acquired an aversion to the reinforcer. These findings demonstrate that amphetamine induced-disruption of goal-directed behavior is mediated by activity at distinct dopamine receptor subtypes and may represent a putative model of the neurochemical processes involved in the loss of voluntary control over behavior.

Repeated administration of psychostimulants such as amphetamine leads to behavioral sensitization and induces the appearance of repetitive and stereotyped behaviors that become more exaggerated following additional drug exposure (Kalivas et al., 1993; Canales et al., 2002; Capper-Loup et al., 2002). Consequently, behavioral sensitization has been used to investigate the neural basis of neuropsychiatric disorders that manifest as inflexible and repetitive patterns of behavior such as drug addiction, OCD and Tourette's (e.g., Canales and Graybiel, 2000; Graybiel and Rauch, 2000; Nestler, 2001; Saka et al., 2004).

Significantly, it has been reported that psychostimulant sensitization also disrupts goal-directed behavior leading to the early and abnormal onset of behaviorally-inflexible habitual responses that are not controlled by their consequences but rather by antecedent stimuli (Nelson and Killcross, 2006; Nordquist et al., 2007). During the early stages of acquisition, instrumental performance is normally sensitive to post-conditioning changes in reward value (Adams and Dickinson, 1981; Dickinson and Balleine, 1994) but as training proceeds, response control is ceded to the habit system and as a consequence becomes less sensitive to changes in reward value (Adams, 1982). However, following pre-training exposure to amphetamine, animals display habit-based instrumental performance that is insensitive to outcome devaluation even with only limited amounts of training (Nelson and Killcross, 2006; Nordquist et al., 2007). This finding supports evidence from lesion studies for a dissociation of neural systems that subserve the performance of voluntary goal-directed actions and reflexive, stimulus-bound habitual responding respectively (Coutureau and Killcross, 2003; Killcross and Coutureau, 2003; Yin et al., 2004, 2005; Naneix et al., 2009; Balleine and O'Doherty, 2010) and suggests that the balance between these two systems is acutely sensitive to manipulations of forebrain dopamine (e.g., Faure et al., 2005; Belin and Everitt, 2008). As imbalances in systems that control instrumental behavior are likely to be involved in the development of habitual drug-taking (Everitt and Robbins, 2005; Hogarth et al., 2013) as well as contribute to the production of involuntary and repetitive behaviors associated with OCD and Tourette's Syndrome (Ridley, 1994; Graybiel and Rauch, 2000; Leckman and Riddle, 2000; Gillan et al., 2011), it is of critical importance to understand the neural mechanisms that underpin the transfer of response control from the goal-directed to the habit system.

The neuropharmacological specificity of this effect, however, remains to be elucidated. Indeed, there is evidence that D_1_ and D_2_ receptor subtypes may have dissociable effects on learning (Beninger and Miller, 1998). For example, it has been shown that Pavlovian approach behavior is attenuated by D_1_ but facilitated by D_2_ antagonists (e.g., Eyny and Horvitz, 2003). These dissociable effects on learning mirror findings that D_1_ and D_2_ receptor subtypes are differentially involved in long-term potentiation (LTP) and depression (LTD) within the striatum: LTP is blocked by D_1_ antagonists (Kerr and Wickens, 2001) but is enhanced by D_2_ antagonists and in D_2_ receptor knock-out mice (Calabresi et al., 1997; Yamamoto et al., 1999). Thus, there are good reasons to assume that the enhancement of S-R habits by prior amphetamine exposure may be mediated by activity at distinct dopamine receptor subtypes.

In the current experiments we sought to reverse amphetamine-induced disruption of goal-directed responding as well as explore the neuropharmacological specificity of this effect by administering both non-selective and selective dopamine antagonists during training. Animals were treated with the non-selective dopamine antagonist α-flupenthixol, the selective D_1_ antagonist SCH 23390 or the selective D_2_ antagonist eticlopride during the acquisition of a moderately trained instrumental response. Subsequently, the reinforcer was devalued by LiCl-induced gastric-malaise and animals' propensity to press the lever was indexed both in extinction and reacquisition. In Experiments 1A–C the dopamine antagonists were administered to animals that had received pre-training exposure to amphetamine and in Experiment 2 the effect of these dopamine antagonists was assessed in non-sensitized animals.

## Materials and methods

### Subjects

Male Lister hooded rats were used in these Experiments (Experiment 1A *n* = 32; Experiment 1B *n* = 32; Experiment 1C *n* = 32; Experiment 2 *n* = 64; Harlan UK Ltd., Bicester, Oxon, UK). At the start of behavioral test animals weighted between 263 and 389 g. Rats were housed in pairs in a climate-controlled vivarium (lights on 8:00 A.M. to 8:00 P.M.) and were tested during the light phase of the cycle. All experimental procedures involving animals and their care were carried out in accordance with the UK Animals Scientific Procedures Act (1986) and were subject to Home Office approval (Project License PPL 30/2158).

### Drugs

For the sensitizing injections (Experiments 1A–C see below) and activity assay (all experiments) *d*-amphetamine sulphate was dissolved in sterile phosphate buffered saline (PBS). Doses of *d*-amphetamine sulphate, 2 mg/kg (sensitizing treatment) and 0.5 mg/kg (activity assay), were calculated as the salt. Alpha-flupenthixol (Experiment 1A) was dissolved in 0.9% physiological saline and administered intraperitoneally (i.p.) 20 min prior to instrumental conditioning at a dose of 0.3 mg/kg. SCH23390 was dissolved in 0.9% physiological saline and administered i.p. 15 min prior to instrumental conditioning at a dose of 0.005 mg/kg. Eticlopride was dissolved in 0.9% physiological saline and administered i.p., 15 min prior to instrumental conditioning at a dose of 0.05 mg/kg (Experiment 1C) and a lower dose of 0.02 mg/kg (Experiment 2). For all drugs, 0.9% saline served as control vehicle solution. All drugs were purchased from Sigma-Aldrich, UK.

### Apparatus

The training apparatus comprised eight chambers (Paul Fray Ltd, Cambridge, UK) measuring 25 × 25 × 22 cm. The chambers were individually housed within sound-attenuating cabinets and were ventilated by low noise fans. Each chamber had three aluminum walls and a clear Perspex front wall. The roof was made of clear Perspex and the floor consisted of 18, 5 mm diameter steel bars spaced 1.5 mm apart centre-to-centre, parallel to the back of the chamber. A recessed magazine that provided access to rewards via a hinged Plexiglas panel was located in the centre of the left-hand wall. The liquid rewards (0.1 ml) could be delivered into the magazine via a peristaltic pump. The reinforcers used were 20% w/v sucrose solution flavored with grape Kool-Aid (0.05% w/v) and 20% w/v maltodextrin solution flavored with cherry Kool-Aid (0.05% w/v) (Cybercandy Ltd., London, UK). Pilot studies indicated that in normal rats these reinforcers were well matched for motivational value but could be easily discriminated. Levers could be inserted to the left and the right of the magazine. A houselight (3W) mounted in the roof provided general illumination. The apparatus and on-line data collection were controlled by means of an IBM-compatible microcomputer equipped with MED-PC software (Med Associates Inc., VT).

### Sensitization

In Experiments 1A–C all rats received *i.p*. injections of 2 mg/kg *d*-amphetamine sulphate once per day for 7 consecutive days. Rats were returned to their home cages immediately after each injection. Over a seven-day injection-free period, animals were reduced to 80% of their *ad libitum* weight, prior to the start of behavioral training. In Experiment 2, animals underwent the same procedure but received i.p. injections of the equivalent volume of saline.

### Behavioral training

Following the sensitization procedure each animal was assigned to one of the eight conditioning chambers, and thereafter was always trained in that chamber. At the start of each session, the house light came on and remained on throughout the session. The house light went out at the end of each session. Behavioral training consisted of three stages: magazine training, instrumental training and devaluation by LiCl.

#### Magazine training

All rats were trained to collect food rewards during two, 30 min magazine training sessions. Half the animals were trained to collect the sucrose solution and the other half the maltodextrin solution (counter-balanced across treatment and devaluation groups). The rewards were delivered on a random time (RT) 60 s schedule by which rewards were delivered, on average, every 60 s.

#### Lever press training and administration of dopamine antagonists

The rats were initially trained to lever press during two sessions on a continuous schedule of reinforcement, with each press producing reward. One lever was inserted into the chamber at the beginning of the session and retracted at the end of the session. Each session continued until the rat had earned 25 reinforcers. In the next three sessions of training, rewards were delivered according to a random interval (RI) 30 s schedule (reward available on average every 30 s and delivered following the next lever press). As current evidence suggests that the critical determinant of sensitivity to outcome devaluation is the degree of exposure to the reinforcer rather than the number of responses made, the number of reinforcers earned during acquisition was strictly controlled (Adams, 1982). Thus In each session, animals earned a total of 40 reinforcers so by the end of training animals had earned a total of 120 rewards on this schedule. This protocol has been shown previously to produce goal-directed responding in controls but accelerated habit formation in amphetamine sensitized animals (Nelson and Killcross, 2006). Prior to each of these lever press training sessions, animals received an i.p. injection of a dopamine antagonist (Drug groups) or the equivalent volume of control vehicle solution (Control group). In Experiments 1A–C half the animals (group Drug) received injections of a dopamine antagonist (α-flupenthixol in 1A, SCH23390 in 1B and eticlopride in 1C) and the other half (Controls) injections of saline. In Experiment 2, 16 animals were administered with α-flupenthixol, 16 with SCH23390, 16 with eticlopride and 16 served as vehicle-injected controls. The experimental conditions are summarized in Table 1.

**Table 1:** **Summary of main experimental findings**.

**Pre-treatment**	**Drug**	**Acquisition**		**Extinction**		**Reacquisition**	
		**LP**	**Mag**	**LP**	**Mag**	**LP**	**Mag**
Amphetamine	α-flupenthixol	↓	–	✓	✓	✓	✓
Amphetamine	SCH23390	↓	–	✓	✓	✓	✓
Amphetamine	Eticlopride	↓	–	✗	✓	✗	✓
Saline	α-flupenthixol	–	–	✓	✓	✓	✓
Saline	SCH23390	↓	–	✓	✓	✓	✓
Saline	Eticlopride	↓	–	?	✓	✓	✓

*In Experiment 1, animals underwent amphetamine sensitization before receiving injections of different dopaminergic antagonists while acquiring an instrumental response. Sensitivity to outcome devaluation was subsequently indexed both in extinction and reacquisition. In Experiment 2, non-sensitized animals underwent identical pharmacological and behavioral procedures. ↓ denotes reduced response rates during acquisition relative to saline injected-controls. ✓ denotes sensitivity and ✗ denotes insensitivity to outcome devaluation during extinction and reacquisition tests. LP denotes lever press behavior and Mag denotes magazine entry behavior*.

### Devaluation by lithium chloride

#### Taste aversion training

After the final day of instrumental lever press training, animals received three days of reward devaluation training with LiCl. On each day the rats were placed in the operant chambers and were given 40 free presentations of the instrumental outcome on an RT 30-s schedule. Immediately after the cessation of each session, the devalued group received a 0.15 M, 10 ml/kg (*i.p*.) injection of LiCl solution (Sigma–Aldrich, UK) and the non-devalued group an injection of the equivalent volume of saline. Taste aversion training was conducted drug-free.

#### Extinction test

24 h after the final session of taste aversion training, animals were placed in the conditioning chambers and received a 10-min, drug-free extinction test conducted in the absence of reward delivery. During this test, lever press performance and magazine entry behavior were assessed.

#### Reacquisition test

In order to confirm that the taste aversion procedure had successfully devalued the outcome for the devalued groups, all animals underwent a 15-min, drug-free reacquisition test. One day after the extinction tests, the animals were placed in the conditioning chambers and lever pressed to earn the instrumental outcome on an RI 30-s schedule.

#### Consumption test (Experiment 1C only)

One day after the reacquisition test, animals were placed in feeding cages and given unrestricted access to the instrumental outcome for 15 min. The test was conducted drug-free.

### Activity assay

To confirm sensitization, all animals were administered a 0.5 mg/kg (*i.p*.) amphetamine challenge before assessment of levels of locomotor activity. These tests occurred immediately following the re-acquisition tests. Activity was monitored using eight chambers (56 cm wide × 39 cm deep × 19 cm high). Activity within each chamber was recorded with pairs of photobeams situated 20 cm apart and 18 cm from the end of the cage connected to a control box (Paul Fray, Cambridge, UK). Each beam break resulted in an incremental count for that chamber and was recorded by an Acorn computer programmed in BBC Basic. Locomotor activity was measured (total number of photobeam breaks) for 30 min.

### Data analysis

Statistical analysis was performed using analysis of variance (ANOVA) with between subject factors of devaluation (devalued versus non-devalued) and drug treatment (either dopamine antagonist or saline). As the standard deviation was proportional to the mean, the extinction data were subject to logarithmic transformations (Howell, 2002). Significant main effects with more than two levels were explored with Tukey *post-hoc* tests.

## Results

### Experiment 1A. The effect of α-Flupenthixol on sensitivity to outcome devaluation after limited training in animals pre-treated with amphetamine

#### Instrumental training

By the end of the three days of RI30 training, all animals had acquired the instrumental response and achieved a stable level of responding. However, α-flupenthixol treatment produced overall lower rates of responding compared to sensitized animals treated with saline. This was confirmed statistically by a main effect of drug [*F*_(1, 28)_ = 7.982, *p* < 0.01] [Mean lever presses per minute (±SEM) AMP + saline group = 10.959 (±1.195); AMP + α-flupenthixol = 7.902 (±0.895)]. However, as the length of each session was determined by the number of reinforcers earned (40 in each) and not time, α-flupenthixol treated animals obtained the same number of reinforcers (120) as controls and hence any differential sensitivity to outcome devaluation observed in the subsequent extinction test cannot be accounted for in terms of differential exposure to the reinforcer. As the critical comparisons at test are between devalued and non-devalued groups within each drug group, it is unlikely that any differences in sensitivity to outcome devaluation are due to these baseline effects. Significantly in this respect, there was neither an effect of intended devaluation (*F* < 1) nor an interaction between drug and devaluation (*F* < 1). In contrast to the depressive effects of α-flupenthixol on lever press acquisition, there was no effect of drug on magazine entry behavior [mean magazine entries per minute (±SEM): AMP + saline group = 5.478 (±1.399); AMP + α-flupenthixol group = 4.642 (±0.974)]. ANOVA yielded no effect of drug (*F* < 1) or devaluation [*F*_(1, 28)_ = 2.224, *p* = 0.145], and no interaction (*F* < 1).

#### Extinction test—lever press performance

**Figure 1:**
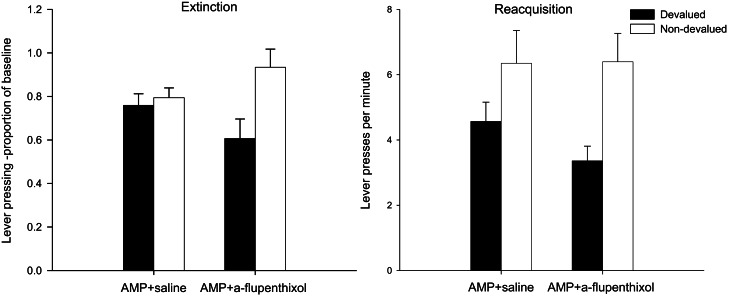
**Effect of α-flupenthixol following amphetamine sensitization on sensitivity of lever pressing to reward devaluation by LiCl-induced nausea.** Mean lever presses per minute as a proportion of baseline (±SEM) in the extinction test (left-hand panel) and lever presses per minute (±SEM) in the rewarded reacquisition test after devaluation by LiCl (devalued—black bars) or no devaluation (non-devalued—white bars).

In order to take account of baseline differences and reduce within subject variability in ANOVA, lever press performance in the extinction test is presented as a proportion of baseline responding. These are presented in the left-hand panel of Figure 1. The suggestion from this figure is that administration of α-flupenthixol during training (group AMP + α-flupenthixol) restored goal-sensitivity as the animals in the devalued group (black bars) showed a selective depression in lever press rates compared to animals in the non-devalued group (white bars). On the other hand the responding of animals exposed to amphetamine before training but administered saline during training (group AMP + saline) appeared to be impervious to the current value of the reinforcer as shown by equivalent rates across the two devaluation groups. There was no effect of drug (*F* < 1) but there was a main effect of devaluation [*F*_(1, 28)_ = 6.598, *p* < 0.05] and critically ANOVA revealed a significant drug × devaluation interaction [*F*_(1, 28)_ = 4.296, *p* < 0.05]. Simple effects analysis of this interaction showed that pre-training amphetamine exposure rendered instrumental performance independent of reward value as there was no devaluation effect in these animals (*F* < 1), but there was an effect of devaluation in the animals treated with α-flupenthixol [*F*_(1, 14)_ = 7.147, *p* < 0.05]. The higher rates of responding in the non-devalued α-flupenthixol-treated rats relative to the non-devalued saline-treated rats may have contributed to the this devaluation effect but simple effects analysis revealed no effect of drug in the non-devalued condition [*F*_(1, 14)_ = 1.98, *p* = 0.17]. As such these results replicate previous findings (Nelson and Killcross, 2006) that pre-training amphetamine exposure leads to accelerated habit formation and suggest that this is an effect reversed by the non-selective dopamine antagonist α-flupenthixol.

#### Extinction test—magazine entry behavior

Analysis of magazine entry behavior during the extinction test revealed a main effect of devaluation [*F*_(1, 28)_ = 12.836, *p* < 0.001] but no effect of drug or interaction between these factors (both *F*s < 1). [Mean magazine entries as a proportion of baseline (±SEM) Devalued group = 0.389 (±0.106); Non-devalued group = 1.09 (±0.249)]. Thus in contrast to lever press performance, magazine entry behavior was sensitive to outcome value irrespective of drug and suggests that the LiCl treatment successfully devalued the value of the instrumental outcome.

#### Reacquisition test—lever press performance

The results of the rewarded reacquisition test confirmed that animals in both devaluation groups had developed an aversion to the reinforcer, as shown in right-hand panel of Figure 1 by reduced lever press rates compared to non-devalued controls. ANOVA revealed an overall effect of devaluation [*F*_(1, 28)_ = 10.036, *p* < 0.01] but this was unaffected by drug group as there was no effect of drug or interaction (both *F*s < 1). Thus, the insensitivity to outcome devaluation in the extinction test observed in the AMP + saline group cannot be attributed to any differential impact of taste aversion training.

#### Reacquisition test—magazine entry behavior

Similarly, magazine entry behavior during the 15-min reacquisition test was sensitive to the changed value of the reinforcer. Both devalued groups performed considerably fewer magazine entries during the test compared to the non-devalued controls [*F*_(1, 28)_ = 11.569 *p* < 0.01] (Mean magazine entries per minute (±SEM) Devalued group = 1.912 (±0.377); Non-devalued group = 3.098 (±0.734). There was no effect of drug [*F*_(1, 28)_ = 1.690, *p* = 0.204] nor a drug × devaluation interaction (*F* < 1).

### Experiment 1B. The effect of SCH23390 on sensitivity to outcome devaluation after limited training in animals pre-treated with amphetamine

#### Instrumental training

All animals acquired the instrumental response but SCH23390 markedly attenuated the rate of responding in animals administered the drug prior to instrumental training. ANOVA yielded a highly significant main effect of drug [*F*_(1, 28)_ = 36.392, *p* < 0.001] [mean lever presses per minute (±SEM) AMP + saline group = 13.967 (±1.435); AMP + SCH23390 group = 6.605 (±0.896)] but no effect of intended devaluation or an interaction between these two factors (both *F*s < 1). However, all animals treated with SCH23390 earned all 120 rewards across the three training sessions and hence had the same exposure to the reinforcer as the animals administered saline during instrumental training. The depressive effects of SCH23390 on responding were restricted to lever pressing, as magazine approach behavior was unaffected by the drug [mean magazine entries per minute (±SEM) AMP + saline group = 5.176 (±0.807); AMP + SCH23390 group = 4.219 (±0.768)]. Statistically, there was no effect of drug [*F*_(1, 28)_ = 1.434, *p* = 0.241], intended devaluation nor an interaction (both *F*s < 1).

#### Extinction test—lever press performance

**Figure 2:**
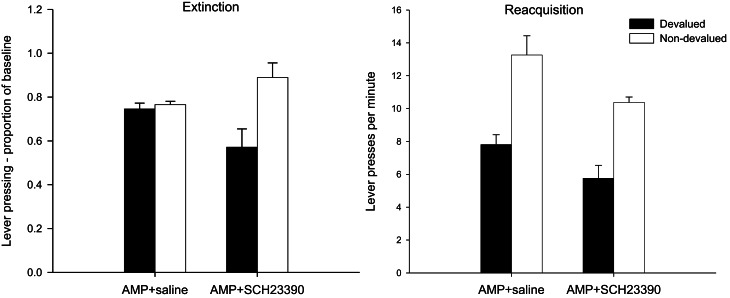
**Effect of SCH23390 following amphetamine sensitization on sensitivity of lever pressing to reward devaluation by LiCl-induced nausea.** Mean lever presses per minute as a proportion of baseline (±SEM) in the extinction test (left-hand panel) and lever presses per minute (±SEM) in the rewarded reacquisition test after devaluation by LiCl (devalued—black bars) or no devaluation (non-devalued—white bars).

The lever press performance of saline injected and SCH23390-treated group during the 10-min extinction as a proportion of their baseline responding is presented in the left-hand panel of Figure 2. Inspection of this figure suggests that the instrumental performance of animals treated with SCH23390 during training was guided by outcome expectancy as the devalued group (black bars) performed fewer lever presses as a proportion of baseline compared to the non-devalued group (white bars). Conversely, the responding of the AMP + saline group in this test was not goal-directed as demonstrated by their failure to show sensitivity to the change in reward value. This description of the data was confirmed statistically by ANOVA which revealed a main effect of devaluation [*F*_(1, 28)_ = 9.157, *p* < 0.01], no effect of drug (*F* < 1) and significantly, a devaluation × drug interaction [*F*_(1, 28)_ =7.146, *p* < 0.05]. Subsequent analysis of this interaction yielded no effect of devaluation in the AMP + saline group (*F* < 1) but devalued and non-devalued performance did differ statistically significantly in animals treated with SCH23390 [*F*_(1, 14)_ = 8.821, *p* < 0.01]. It is possible that the higher rates of responding in the SCH23390 non-devalued group may have contributed to the devaluation × drug interaction but simple effects found no evidence that there was an effect of drug in the non-devalued condition [*F*_(1, 14)_ = 2.47, *p* = 0.13]. These findings suggest that the D_1_ receptor antagonist SCH23390 disrupted the more rapid onset of behavioral autonomy seen after sensitization with amphetamine.

#### Extinction test—magazine entry behavior

In contrast, magazine performance during the extinction test was sensitive to the changed value of the reinforcer in both drug groups [mean magazine entries as a proportion of baseline (±SEM) Devalued group = 0.495 (±0.117); non-devalued group = 1.411 (±0.275)]. Indeed, ANOVA revealed only a main effect of devaluation [*F*_(1, 28)_ = 18.521, *p* < 0.001], no effect of drug (*F* < 1) nor an interaction [*F*_(1, 28)_ = 1.587, *p* = 0.218].

#### Reacquisition test—lever press performance

The effectiveness of the taste aversion training in devaluing the instrumental outcome is further supported by analysis of lever press rates performed in the rewarded reacquisition test shown in the right-hand panel of Figure 2. The impression from this figure is that all animals were averted from the reinforcer, irrespective of drug treatment, and hence pressed the lever at lower rates compared to the non-devalued controls. Statistical analysis by ANOVA revealed a highly significant main effect of devaluation [*F*_(1, 28)_ = 25.112, *p* < 0.001] as well as a main effect of drug [*F*_(1, 28)_ = 6.031, *p* < 0.05] reflecting overall lower response rates in the SCH23390 group, but the level of devaluation in these animals was comparable to that of the AMP + saline animals as there was no drug × devaluation interaction (*F* < 1).

#### Reacquisition test—magazine entry behavior

Magazine entry behavior was equally sensitive to outcome value in both drug groups during the reacquisition test [Mean magazine entries per minute (±SEM): Devalued group = 3.983 (±1.408); Non-devalued group = 8.036 (±1.348)]. Statistically, there was an overall effect of devaluation [*F*_(1, 28)_ = 10.524, *p* < 0.01] but no effect of drug (*F* < 1) nor an interaction [*F*_(1, 28)_ = 1.322, *p* = 0.26]. Thus in contrast to lever press performance in the reacquisition test, magazine approach behavior was unaffected by SCH23390.

### Experiment 1C. The effect of eticlopride on sensitivity to outcome devaluation after limited training in animals pre-treated with amphetamine

#### Instrumental training

Both drug groups acquired the instrumental response, albeit at different rates. Eticlopride greatly reduced the rate of responding compared to animals given saline during training. Statistically, ANOVA revealed a highly significant main effect of drug [*F*_(1, 28)_ = 34.205, *p* < 0.001] [mean lever presses per minute (±SEM) AMP + saline group = 12.411 (±1.005); AMP + eticlopride group = 7.0122 (±0.795)] but no effect of devaluation group or an interaction (both *F*s < 1). As session length was determined by number of rewards earned (40 per session) rather than time, all the animals in the Eticlopride group earned the 120 rewards over the three sessions. Conversely, eticlopride had no impact on magazine entry behavior as there was no effect of drug, devaluation or an interaction (all *F*s < 1) [mean magazine entries per minute (±SEM): AMP + saline group = 4.311 (±0.610); AMP + eticlopride group = 5.055 (±0.834)].

#### Extinction test—lever press performance

**Figure 3:**
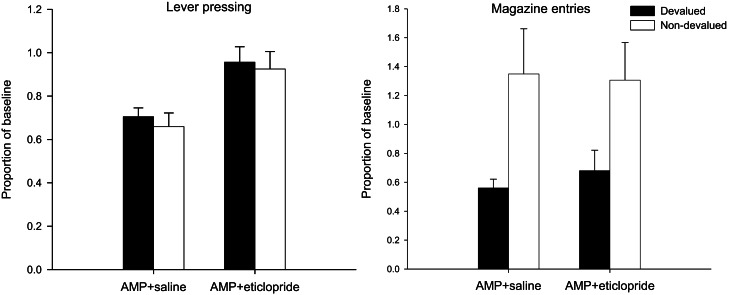
**Effect of eticlopride following amphetamine sensitization on sensitivity of lever pressing (left-hand panel) and magazine entry (right-hand panel) to reward devaluation by LiCl-induced nausea.** Mean lever presses per minute and mean magazine entries per minutes as a proportion of baseline (±SEM) in the extinction test after devaluation by LiCl (devalued—black bars) or no devaluation (non-devalued—white bars).

The mean lever presses per minute in the critical extinction test are presented in the left-hand panel of Figure 3. It is clear from this figure that none of the animals, irrespective of drug group, was sensitive to the changed value of the reinforcer as both devalued groups responded at equivalent rates to the non-devalued controls. This was confirmed statistically as there was no effect of devaluation (*F* < 1) and no interaction between drug and devaluation factors (*F* < 1). Eticlopride therefore failed to reverse the effect of pre-training amphetamine exposure on goal-sensitivity after limited training and responding in both groups was habitual even after limited training. However, ANOVA did reveal a highly significant main effect of drug [*F*_(1, 28)_ = 15.578, *p* < 0.001], reflecting overall higher rates of responding as a proportion of baseline in the eticlopride group. As the extinction test was conducted drug-free and the data were analyzed as a proportion of baseline, the effect of drug at test may in part reflect the lower rates of responding seen during acquisition under eticlopride. However, the finding that eticlopride-treatment led to reduced responding during acquisition but failed to abolish the enhancement of S-R habits by amphetamine sensitization suggests that the restoration of goal-sensitivity by α-flupenthixol and SCH23390 (Experiments 1A,B, see above) cannot be attributed to their depressive effects on response rates during acquisition alone.

#### Extinction test—magazine entry behavior

Despite the insensitivity of lever pressing to outcome devaluation, it is clear from the right-hand panel of Figure 3 that magazine entry behavior in both devalued groups was reduced compared to non-devalued controls. Statistical analysis revealed only an effect of devaluation [*F*_(1, 28)_ = 10.576, *p* < 0.01] and no effect of drug nor an interaction (both *F*'s < 1). Thus the demonstration that lever press performance in the extinction test was under the control of S-R habits, whereas magazine approach behavior was guided by outcome value, indicates that the LiCl treatments successfully devalued the instrumental outcome.

#### Reacquisition test—lever press performance

**Figure 4:**
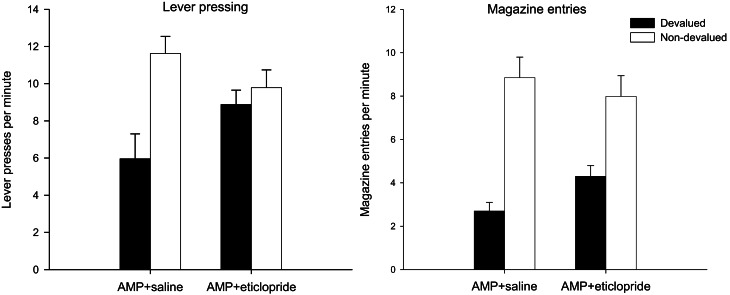
**Effect of eticlopride following amphetamine sensitization on sensitivity of lever pressing (left-hand panel) and magazine entry (right-hand panel) reacquisition after reward devaluation by LiCl-induced nausea.** Mean lever presses per minute and mean magazine entries per minutes as a proportion of baseline (±SEM) in the rewarded reacquisition test after devaluation by LiCl (devalued—black bars) or no devaluation (non-devalued—white bars).

The results of the rewarded reacquisition test revealed an intriguing dissociation in performance between the two drug groups. The saline treated animals averted from the reinforcer showed a clear devaluation effect: this is consistent with the direct punishment of S-R habits by the presentation of the nausea-inducing reinforcer and with previous findings that pre-training amphetamine exposure promotes lever press performance that is insensitive to outcome devaluation in extinction but not in reacquisition (see Nelson and Killcross, 2006). However, as is clear from the left-hand panel of Figure 4, the devalued animals in the eticlopride group pressed the lever at comparable rates to the non-devalued controls even though responding was reinforced with the reward that had been previously paired in these animals with gastric malaise. This description of the data was supported statistically by ANOVA which revealed a main effect of devaluation [*F*_(1, 28)_ = 10.384, *p* < 0.01], no effect of drug (*F* < 1), but crucially a significant interaction between these two factors [*F*_(1, 28)_ = 5.472, *p* < 0.05). Subsequent analysis of this interaction with simple effects confirmed that saline-treated animals had acquired an aversion to the reinforcer and could use this representation to guide instrumental performance when presented with the consequences of their actions in reacquisition as there was a highly significant effect of devaluation in these animals [*F*_(1, 14)_ = 12.171, *p* < 0.01]. There was no such effect in the eticlopride-treated animals (*F* < 1). This can be taken as evidence that instrumental performance in eticlopride treated animals was completely impervious to reward value and had become compulsive. However, it is possible that this insensitivity arose from a failure of the taste aversion training.

#### Reacquisition test—magazine entry behavior

Significantly, analysis of magazine entry behavior during the rewarded reacquisition test suggests that all animals, regardless of drug treatment, had acquired an aversion to the reinforcer. The mean magazine entries per minute in this test are displayed in the right-hand panel of Figure 4 and in stark contrast to the lever press data reviewed above, magazine approach behavior was sensitive to reward value in both drug groups. ANOVA yielded no effect of drug (*F* < 1) and a highly significant effect of devaluation [*F*_(1, 28)_ = 45.598, *p* < 0.001). The suggestion from the right hand-panel of Figure 4 is that the devaluation effect may have been slightly attenuated in the eticlopride group but there was no statistical evidence for this as the interaction failed to reach significance [*F*_(1, 28)_ = 2.743, *p* = 0.109].

#### Consumption test

In order to confirm that the differential sensitivity of lever press to reward value observed in the reacquisition test could not be explained in terms of any failure of eticlopride-treated animals to acquire an aversion to the reinforcer, all animals were given free access to the instrumental outcome and consumption was measured over a 15-min period. Results of this consumption test revealed that all animals averted from the reinforcer consumed statistically significantly less of the instrumental outcome compared to the non-devalued controls (mean consumption in ml (±SEM): Devalued AMP + saline group = 3.263 (±0.549); Non-devalued AMP + saline group = 6.175 (±0.966); Devalued AMP + eticlopride group = 4.15 (±0.711); Non-devalued AMP + eticlopride group = 7.00 (±0.603). ANOVA revealed a main effect of devaluation [*F*_(1, 28)_ = 15.776, *p* < 0.001] and a non-significant trend toward marginally higher overall consumption in eticlopride-treated animals [*F*_(1, 28)_ = 1.393, *p* = 0.248]. Critically, the devaluation effect was unaffected by drug as there was no interaction between these two factors (*F* < 1). Coupled with evidence that magazine entry behavior was sensitive to outcome value in both the extinction and reacquisition tests, the results of the consumption test confirm that all animals had acquired an aversion to the reinforcer and hence the effects of eticlopride on the sensitivity of lever pressing to reward value cannot be accounted for in terms of any ineffectiveness of the LiCl devaluation treatments.

### Experiment 2. The effect of α-Flupenthixol, SCH23390, and eticlopride on the sensitivity to outcome devaluation after limited training in non-sensitized animals

#### Instrumental training

As expected, the dopamine antagonists reduced the rate of responding and this effect was particularly marked in animals treated with SCH23390 and eticlopride [mean lever presses per minute (±SEM) Saline group = 10.693 (±1.033); α-flupenthixol group = 8.504 (±1.249); SCH23390 group = 6.551 (±0.899); Eticlopride group = 5.819 (±1.245)]. Despite the reduction in the rate of responding, all animals earned 120 reinforcers across the three sessions. This description of the data was confirmed by ANOVA which revealed a main effect of drug [*F*_(3, 56)_ =7.397, *p* < 0.001] but no effect of intended devaluation nor an interaction between these factors (both *F*'s < 1). Subsequent *post-hoc* analysis with Tukey tests confirmed that both SCH23390- (*p* < 0.01) and eticlopride- (*p* < 0.001) treated animals responded at lower rates than saline treated animals. However, magazine entry behavior was unaffected by any of these factors as there was no effect of drug, intended devaluation or interaction [highest *F*_(1, 56)_ = 1.947, *p* = 0.168] [Mean magazine entries per minute (±SEM): Saline group = 4.906 (±0.576); α-flupenthixol group = 4.575 (±0.634); SCH23390 group = 4.777 (±0.807); Eticlopride group = 3.809 (±0.678)].

#### Extinction test—lever press performance

**Figure 5:**
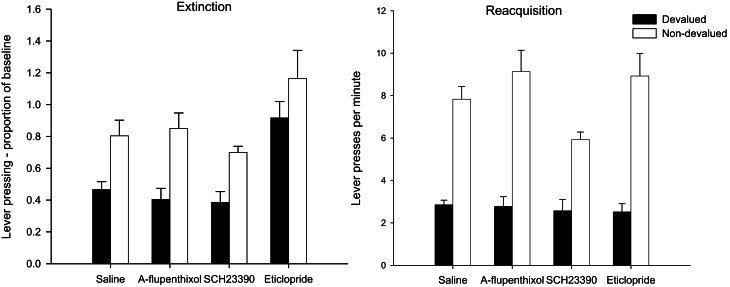
**Effect of α-flupenthixol, SCH23390 and eticlopride on sensitivity of lever pressing to reward devaluation by LiCl-induced nausea.** Mean lever presses per minute as a proportion of baseline (±SEM) in the extinction test (left-hand panel) and lever presses per minute (±SEM) in the rewarded reacquisition test after devaluation by LiCl (devalued—black bars) or no devaluation (non-devalued—white bars).

The left-hand panel of Figure 5 displays the lever press performance in the extinction test following devaluation by LiCl. Inspection of this figure suggests that saline controls and animals given SCH23390 and α-flupenthixol during training were goal-directed as animals averted from the reinforcer showed a marked suppression in lever press performance compared to non-devalued control animals. The suggestion from this figure is that the devaluation effect may have been attenuated in animals treated with eticlopride. However, ANOVA only revealed a main effect of devaluation [*F*_(1, 56)_ = 24.317, *p* < 0.001] and no interaction between drug and devaluation (*F* < 1). There was an effect of drug [*F*_(3, 56)_ = 10.708, *p* < 0.001] due to overall higher rates of responding in the eticlopride treated animals. *Post-hoc* Tukey tests revealed that the eticlopride-treated animals pressed at significantly higher rates than all other animals (all *ps* < 0.01).

#### Extinction test—magazine entry behavior

Analysis of magazine entry behavior during the 10-min extinction test suggests that the LiCl treatment successfully devalued the outcome for all animals as there was a main effect of devaluation [*F*_(1, 56)_ = 9.661, *p* < 0.01] [mean magazine entries as a proportion of baseline (±SEM) Devalued group = 0.443 (±0.059); Non-devalued group = 0.769 (±0.083)]. However, this effect was unaffected by drug group as there was no main effect of drug or an interaction (both *F*s < 1).

#### Reacquisition test—lever press performance

The results of the rewarded reacquisition test presented in the right-hand panel of Figure 5 confirmed that all animals had acquired an aversion to the reinforcer. ANOVA yielded a highly significant effect of devaluation [*F*_(1, 56)_ = 138.828, *p* < 0.001] as well as an effect of drug [*F*_(3, 56)_ = 2.774, *p* < 0.05] reflecting lower responding in the SCH23390 group. *Post-hoc* Tukey tests showed that the rate of responding in SCH23390 treated animals differed only from that of α-flupenthixol group (*p* < 0.05). The overall lower responding in the SCH23390-treated animals and in particular the non-devalued SCH23390-treated animals, would account for a marginal significant drug × devaluation interaction [*F*_(3, 56)_ = 2.512, *p* = 0.068]. Nevertheless it is evident from the right-hand panel of Figure 5 that all devalued groups had acquired a robust aversion to the instrumental outcome and consequently suppressed lever press responding during the rewarded test.

#### Reacquisition test—magazine entry behavior

This impression was also confirmed by analysis of magazine approach behavior during the rewarded reacquisition test, with all animals in the devalued groups performing fewer magazine entries compared to the non-devalued controls [*F*_(1, 56)_ = 28.010, *p* < 0.001]. There was also a main effect of drug [*F*_(3, 56)_ = 6.521, *p* < 0.001] as the α-flupenthixol treated animals had higher rates of magazine approach behavior (*p* < 0.01) but this heightened responding did not impact on sensitivity of magazine entry behavior to outcome devaluation as there was no drug x devaluation interaction (*F* < 1).

### Activity assay

**Figure 6:**
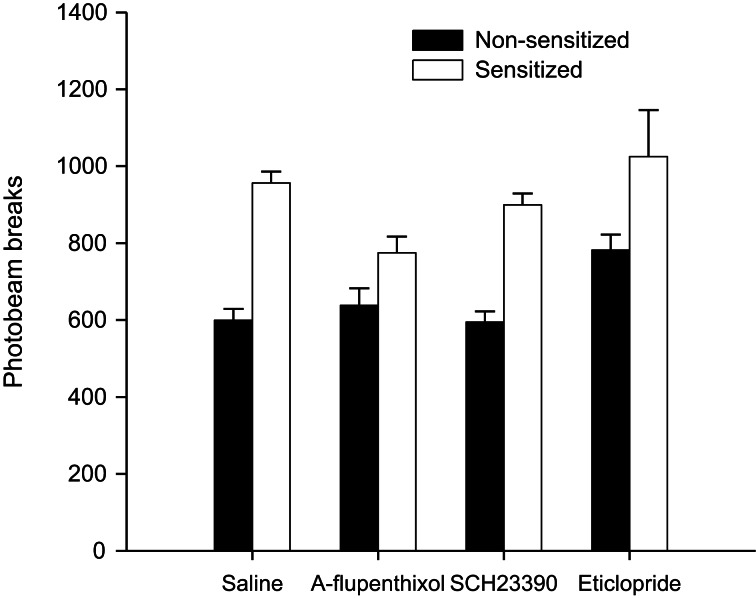
**Activity assay—total mean photobeam breaks following a 0.5 mg/kg amphetamine challenge in sensitized (white bars) and non-sensitized (black bars) animals treated with saline, α-flupenthixol, SCH23390 or eticlopride during training**.

In order to confirm the presence of sensitization in amphetamine pre-treated animals, all animals were administered a 0.5 mg/kg amphetamine challenge allowing between subject comparisons of the locomotor activating effects of amphetamine in sensitized (Experiments 1A–C) and non-sensitized animals (Experiment 2). As is clear from Figure 6 animals with prior experience of amphetamine showed elevated levels of locomotor activity compared to drug-naïve animals. ANOVA with between-subject factors of sensitization (sensitized with amphetamine or non-sensitized drug-naïve animals) and drug administered during training (saline, α-flupenthixol, SCH23390, or eticlopride) yielded a highly significant effect of sensitization [*F*_(1, 144)_ = 48.909, *p* < 0.001] but also an effect of drug [*F*_(3, 144)_ = 4.798, *p* < 0.01] due to higher locomotor activity in response to the amphetamine challenge in all animals treated with eticlopride during training. There was, however, no interaction between sensitization and drug [*F*_(3, 144)_ = 1.702, *p* = 0.169]. These results confirm that the amphetamine pre-treatment had successfully sensitized animals to amphetamine and provide indirect evidence that antagonism with the D_2_ antagonist eticlopride enhances the locomotor activating effects of amphetamine irrespective of prior experience with amphetamine.

## Discussion

The experiments reported here examined the effects of both non-selective and selective dopamine antagonists on instrumental performance in a reinforcer devaluation task either in animals given pre-training exposure to amphetamine (Experiments 1A–C) or in non-sensitized animals. Significantly, the experiments replicated our previous finding that pre-training exposure to amphetamine renders instrumental performance autonomous of the current value of the reinforcer even after limited training (Nelson and Killcross, 2006). The results demonstrated that accelerated habit formation seen after amphetamine sensitization is reversed by D_1_, but enhanced by D_2_ receptor antagonists. Furthermore, these experiments provided considerable insights into the role of D_1_ and D_2_ receptor subtypes in mediating instrumental learning generally as well as susceptibility to devaluation procedures in sensitized and non-sensitized animals.

Before considering test performance, it is important to address the effect of the various dopamine antagonists on acquisition of the instrumental response. Consistent with previous reports from operant procedures (e.g., Tombaugh et al., 1979; Wise and Schwartz, 1981) the administration of dopamine antagonists during training severely retarded the rate of responding in both sensitized and non-sensitized animals. It is important to note that, despite these reduced rates, all animals earned the same number of reinforcers during training as session length was limited by the number of reinforcers earned and not time (Adams, 1982). In the current experiments there was some evidence of a dissociation at the receptor subtype level between the performance of instrumental responses under drug and their expression in drug-free tests. As dopamine has been implicated in various non-associative factors such as motivation, attention and sensorimotor control that contribute to learning, any effects of dopamine antagonism that are restricted to the performance of an instrumental response under drug can be attributed to these non-associative factors. However, if effects of dopaminergic manipulations are seen on the drug-free expression of learned instrumental responses, for example in the current experiments in the extinction and reacquisition tests, then this can be taken as evidence to suggest that a dopaminergic agent may have modulated the course of associative learning.

Here, the non-selective antagonist α-flupenthixol and the selective D_2_ antagonist eticlopride reduced the rate of instrumental responding during acquisition, but at test the response rates, expressed as a proportion of these reduced baseline rates, were actually higher relative to saline controls. This recovery of responding in the drug-free extinction text indicates that antagonism of D_2_ receptors may have disrupted the performance of that response during acquisition and not the expression of that learning in the drug-free extinction test. Moreover, these animals showed comparable rates of responding to saline controls in the drug-free reacquisition test. This could similarly be taken as evidence to suggest that these drugs disrupted the performance i.e., led to reduced rates of responding during acquisition but not the subsequent drug-free expression of instrumental conditioning in reacquisition. Dopamine activity in the nucleus accumbens, a structure containing the highest concentration of D_2_ receptors in the rat brain (Bentivoglio and Morelli, 2005), has been widely implicated in the reinforcing and motivational properties of both natural rewards and drugs of abuse (e.g., Hernandez and Hoebel, 1988; Mark et al., 1994; Wyvell and Berridge, 2000). Thus the disruptive effects of agents selectively and non-selectively targeting D_2_ receptors on the performance but not the drug-free expression of the instrumental response may have been due to decreased motivation associated with these drugs. However, it is equally possible, the reduced rate of responding could have arisen as a result of the profound motor impairments typically produced by D_2_ antagonists (e.g., Fowler and Liou, 1994). Whether the disruption was caused by motivational or motor factors or a combination of the two, D_2_ receptor antagonism appeared to impair the performance but not the drug-free expression of the instrumental response in the current experiments. Conversely, D_1_ antagonism by SCH23390 not only affected the performance during training but it also reduced the expression of learned instrumental responses at reacquisition. The test was conducted five days after the last SCH23390 treatment and hence the reduced rate of responding may not be accounted for solely in terms of drug induced motivational or sensorimotor deficits but of course these factors cannot be entirely discounted. The results are consistent with previous reports of disruption to operant responding by SCH23390 (e.g., Nakajima, 1986; Sharf et al., 2005) and suggest that D_1_ receptors may be involved in the associative learning as well as other processes underpinning instrumental responding.

In stark contrast to the effects of dopaminergic drugs on instrumental performance, antagonism of dopaminergic systems failed to impact on magazine approach behavior (but see Choi et al., 2005). Both during acquisition and at test there was no effect of the various dopaminergic agents used in the current experiments on magazine entry behavior. Furthermore in line with previous evidence, magazine approach behavior remained sensitive to outcome devaluation even when instrumental performance (see below) was impervious to changes in reward value (Nelson and Killcross, 2006). Thus the deficits in instrumental performance observed cannot *simply* be attributable to motoric dysfunction as any drug induced motor impairment would presumably impact on magazine approach behavior as well as lever pressing. To the extent that magazine approach behavior in a free operant procedure depends on Pavlovian contingencies, these findings provide yet further evidence that Pavlovian and instrumental conditioning can be subserved by distinct psychological and neural processes (e.g., Holland, 1998; Dickinson et al., 2000; Corbit et al., 2001).

As expected, animals that were not exposed to amphetamine prior to training showed normal sensitivity to outcome devaluation after limited training. The administration of the dopamine antagonists α-flupenthixol and SCH23390 during training had no impact on this sensitivity; it was neither enhanced nor attenuated by these drugs. Eticlopride treatment, however, appeared to reduce sensitivity to the changed value of the reinforcer after taste aversion as evidenced by comparable rates of responding across the two devaluation groups. As there was no statistical evidence for this effect, any inferences from Experiment 1C about the role of D_2_ receptors in the control of goal-directed behavior in normal animals would be premature.

Nevertheless, the results from the reinforcer devaluation task in animals with prior exposure to amphetamine (Experiments 1A–C) furnish unequivocal evidence for distinct roles of D_1_ and D_2_ receptor subtypes in the control of behavior by goal-directed actions and S-R habits. In a replication of our previous findings, animals given pre-training exposure to amphetamine and saline during training showed accelerated habit formation as they failed to alter lever press performance in response to the changed value of the reinforcer. The performance in the reinforcer devaluation task of sensitized animals treated with either the non-selective dopamine antagonist α-flupenthixol or the D_1_ antagonist SCH23390 during training was not autonomous of the current value of the reinforcer as these animals showed a selective depression in lever press rates compared to non-devalued controls. Thus the instrumental performance of these animals mirrors that of normal animals after limited training and suggests response control was by goal-directed A-O associations. Given that α-flupenthixol is a non-selective dopamine antagonist that acts at both D_1_ and D_2_ receptors it is perhaps noteworthy that its effects in the current study were comparable to those seen with the selective D_1_ antagonist SCH23390 and not the selective D_2_ antagonist eticlopride. This would suggest that blockade of D_1_ receptors by α-flupenthixol was sufficient to reverse amphetamine-induced disruption of goal-directed behavior. Consistent with this profile of action, amphetamine-induced disruption of conditional discrimination performance is attenuated by acute treatment with both selective D_1_ antagonists and α-flupenthixol but not D_2_ antagonists (Dunn et al., 2005; Dunn and Killcross, 2006).

The finding, however, that instrumental responding in animals given eticlopride was impervious to the current value of the reinforcer suggests these animals' instrumental performance remained stimulus-bound and governed by S-R habits. The differential sensitivity to outcome devaluation procedures cannot be attributed to impaired acquisition, as responding in all animals was depressed during acquisition irrespective of the antagonist administered. Similarly, all animals acquired an aversion to the reinforcer as evidenced by the marked sensitivity of magazine approach to outcome value in the extinction tests. The consumption test in Experiment 1C similarly confirmed that eticlopride-treated animals had acquired an aversion to the reinforcer and were able to inhibit consummatory behavior. Furthermore, the magazine entry data suggest that the eticlopride treated animals were able, under certain circumstances, to inhibit specific responses. It is not entirely possible to preclude response perseveration as an explanation of the results but the sensitivity of magazine approach to changes in reward value suggests that the insensitivity of lever pressing to outcome devaluation in these animals is unlikely to be entirely attributable to general response perseveration. Although the results of the activity test indicated elevated locomotor activity in eticlopride-treated animals in response to an amphetamine challenge compared to other animals, the sensitivity of magazine entry behavior renders any interpretation of lever press performance in terms of hyperactivity unlikely. The results are therefore specific to an effect on lever pressing and demonstrate that the accelerated habit formation following amphetamine exposure is prevented by D_1_ but not D_2_ receptor antagonism. Indeed, this parallels good evidence that the development of sensitization to the locomotor activating effects of amphetamine is also blocked by D_1_ antagonists. These effects have been observed systemically (Vezina and Stewart, 1989) and with local infusions of SCH23390 into both the VTA and substantia nigra pars reticulata (Stewart and Vezina, 1989; Vezina, 1996). Similarly, D_1_ receptor knock-out mice fail to develop behavioral sensitivity to amphetamine (Karper et al., 2002; McDougall et al., 2005) and a fMRI study supports the suggestion that D_1_ receptors are responsible for amphetamine-mediated neurochemical changes and that D_1_ antagonists inhibit this response to amphetamine (Dixon et al., 2005). Thus the current findings concur with reports of D_1_ receptor modulation of the neurochemical and locomotor response to amphetamine and extend them to include a further behavioral response; enhanced habit formation.

However, eticlopride administered during training failed to reverse the accelerated formation of S-R habits induced by pre-training amphetamine exposure. This finding is consistent with evidence that D_2_ antagonism can actually enhance the behavioral and neurochemical effects of amphetamine. For example, the blockade of D_2_ receptors in the VTA produces persistent elevation of the locomotor activating effects of amphetamine (Tanabe et al., 2004). Indeed, in the current experiments systemic administration of eticlopride during training appeared to heighten the potentiation of locomotor activity by amphetamine in both sensitized and non-sensitized animals in the activity test following a drug challenge. Sulpiride, which has high affinity for D_2_ receptors, has been shown to enhance the augmentative effects of amphetamine on extracellular striatal dopamine levels measured by *in vivo* microdialysis (Jaworski et al., 2001). Similarly, fMRI measurement of changes in rat brain activation following amphetamine administration shows that pre-treatment with sulpiride facilitates the response elicited by amphetamine (Dixon et al., 2005). Furthermore, the finding that the instrumental performance of animals treated with eticlopride was completely independent of goal-value during the reacquisition test also suggests that antagonism of D_2_ receptors enhanced the effect of pre-training exposure to amphetamine on the sensitivity of a moderately trained instrumental response to outcome devaluation. The amphetamine-sensitized animals treated with eticlopride clearly had a representation of the devalued outcome as they inhibited magazine entry responses and when given the opportunity consumed less of the outcome compared to controls, but they failed to use this representation to guide instrumental responding. Instrumental performance under the control of S-R habits, whether engendered by overtraining or amphetamine exposure, is normally sensitive to outcome value in re-acquisition and thus the insensitivity of eticlopride-treated animals in the reacquistion test in Experiment 1C is novel and can be taken as evidence of dysfunctional habit learning characteristic of compulsions. By definition, compulsive behavior is carried out repetitively and persists despite adverse consequences. Significantly, there is evidence that abnormal D_2_ receptor binding may be involved in psychopathologies characterized by compulsive behavior. For example, PET scans have revealed low D_2_ receptor availability in drug abusers (Wang et al., 1999; Volkow et al., 1999, 2001, 2007) and single photon emission computerized tomography (SPECT) has shown reduced D_2_ receptor binding in OCD patients (Denys et al., 2004). The current results are consistent with these reports and suggest that sensitization of dopaminergic systems coupled with antagonism of D_2_ receptors may lead to maladaptive habitual behavior that is compulsive. As such the paradigm developed here could serve as model of the neurochemical changes that accompany the loss of voluntary control over behavior associated with drug addiction and neuropsychiatric disorders such as OCD and Tourette's Syndrome.

The finding of opposing roles of D_1_ and D_2_ receptors in the transition from action to habit and compulsion in the experiments presented here is consistent with previous reports that antagonism of D_1_ receptors disrupts, but D_2_ receptor blockade facilitates, learning in a variety of Pavlovian conditioning paradigms (Smith et al., 1997; Horvitz, 2001; Eyny and Horvitz, 2003; Yue et al., 2004; Cassaday et al., 2005). The demonstration here of dissociable effects of D_1_ and D_2_ receptor antagonism on instrumental learning and the sensitivity of that learning to outcome devaluation is, however, novel. Moreover, it is consistent with evidence that activity at D_1_and D_2_ receptor subtypes can exert opposing effects on dendritic excitability and neuroplasticity within the striatum that in turn may facilitate or inhibit appropriate action selection (Surmeier et al., 2007; Gerfen and Surmeier, 2011). This differential involvement in striatal synaptic plasticity may therefore underlie the effects on learning seen here and more generally accelerated habit formation after sensitization (Gerdeman et al., 2003).

More broadly, the current findings have implications for our understanding of the role of dopamine and activity at different dopamine receptor subtypes in modulating behavioral flexibility. These data provide evidence of D_1_ receptor involvement in the transition from flexible goal-directed action to inflexible stimulus-driven habits and raise the possibility that antagonism of D_1_ receptors would reinstate goal-directed behavior in overtrained rats. Similarly, D_1_ receptor knock-out mice may fail to develop goal-insensitive habitual responding. Conversely, antagonism of D_2_ receptors appears to exert the opposite effect and render instrumental behavior completely insensitive to changes in outcome value. Thus, antagonism of D_1_ but not D_2_ receptors can produce flexible goal-directed behavior when it would otherwise be inflexible.

### Conflict of interest statement

The authors declare that the research was conducted in the absence of any commercial or financial relationships that could be construed as a potential conflict of interest.
